# Prenatal stress from trawl capture affects mothers and neonates: a case study using the southern fiddler ray (*Trygonorrhina dumerilii*)

**DOI:** 10.1038/srep46300

**Published:** 2017-04-12

**Authors:** L. Guida, C. Awruch, T. I. Walker, R. D. Reina

**Affiliations:** 1 School of Biological Sciences, Monash University, Clayton, Victoria 3800, Australia; 2 CESIMAR (Centro Para el Estudio de Sistemas Marinos) – CENPAT- CONICET, Puerto Madryn, Chubut U9120ACD, Argentina; 3 School of Biological Sciences, University of Tasmania, Hobart, Tasmania 7001, Australia

## Abstract

Assessing fishing effects on chondrichthyan populations has predominantly focused on quantifying mortality rates. Consequently, sub-lethal effects of capture stress on the reproductive capacity of chondrichthyans are largely unknown. We investigated the reproductive consequences of capture on pregnant southern fiddler rays (*Trygonorrhina dumerilii*) collected from Swan Bay, Australia, in response to laboratory-simulated trawl capture (8 h) followed immediately by air exposure (30 min). Immediately prior to, and for up to 28 days post trawling, all females were measured for body mass (BM), sex steroid concentrations (17-β estradiol, progesterone, testosterone) and granulocyte to lymphocyte (G:L) ratio. At parturition, neonates were measured for total length (TL), BM and G:L ratio. Trawling reduced maternal BM and elevated the G:L ratio for up to 28 days. Trawling did not significantly affect any sex steroid concentrations relative to controls. Neonates from trawled mothers were significantly lower in BM and TL than control animals, and had an elevated G:L ratio. Our results show that capture of pregnant *T. dumerilii* can influence their reproductive potential and affect the fitness of neonates. We suggest other viviparous species are likely to be similarly affected. Sub-lethal effects of capture, particularly on reproduction, require further study to improve fisheries management and conservation of chondrichthyans.

Populations of many chondrichthyan species have been reduced by the effects of fishing^1^,^2^, with an estimated one quarter of all species worldwide appearing on the IUCN Red List of Threatened Species^3^. Chondrichthyans are often incidentally caught in commercial and recreational fisheries as bycatch (discarded) or byproduct (retained). To conserve and manage chondrichthyan populations it is essential that we understand their ability to survive human impacts to ensure that populations contribute to future generations.

The effects of capture, handling and release techniques on chondrichthyan are predominantly assessed by measuring the more immediate and short-term (usually a few days) physiological responses to acute stress, which may or may not result in immediate or delayed mortality^4^,^5^,^6^,^7^,^8^,^9^. In contrast, the sub-lethal effects of capture stress have largely been ignored in chondrichthyan studies^10^. However, a significant, longer-term and ecologically important impact of fisheries capture may be that of altered or impaired reproduction.

Alterations to reproductive physiology are amongst the most frequently observed consequences of stress across a range of vertebrae taxa^11^,^12^. Stress is known to influence maternal body condition^13^,^14^, cause premature parturition^15^ and influence offspring size, behaviour and immunocompetence^16^,^17^,^18^. Reproductive consequences of stress are largely mediated by the maternal endocrine response which regulates energy allocated to self-maintenance relative to reproduction itself^19^. Little is known about endocrine regulation of reproduction in chondrichthyans, including the roles of the primary sex steroids 17β-estradiol (E_2_), progesterone (P_4_) and testosterone (T), but it appears structurally and functionally similar to other vertebrates^20^. However, because of the diversity of reproductive strategies within chondrichthyan viviparity, ranging from lecithotrophic viviparity to placental viviparity, it is difficult to generalise the roles of hormones regulating reproductive events^20^. For several species examined so far, E_2_ is generally associated with follicular development and yolk uptake followed by increases in P_4_ at the onset of ovulation. Increased P_4_ is thought to maintain pregnancy until nearing parturition, at which point P_4_ starts to decline^21^,^22^,^23^,^24^,^25^. The role of T is less well understood, although it is thought to regulate final maturation of the ovarian follicles, mating behaviour^25^ and possibly the control of embryonic diapause^23^. It is not known how acute or chronic stress influences maternal sex steroid synthesis in chondrichthyans, but alterations to their circulating concentrations may reveal their effect on reproductive investments such as ovulation and follicular development^26^,^27^,^28^.

In reproductively active females, energy is often directed away from immune function and redistributed towards the higher energetic demands of reproduction, resulting in an immunosuppressed state^29^,^30^,^31^, which can be identified by an increase in the proportion of circulating granulocytes^32^. Because of the increased energetic cost to mount an immune response, immune challenges during reproduction can further strain energetic resources and reduce maternal body mass, which is an indicator of chronic stress^31^,^33^. Measurement of the granulocyte to lymphocyte (G:L) ratio is frequently used to measure stress in a range of vertebrate taxa and its sustained elevation can be indicative of chronic stress and impaired immune function^34^,^35^. In chondrichthyans, both the draughtboard sharks, *Cephaloscyllium laticeps*^8^, and sparsely-spotted stingarees, *Urolophus paucimaculatus*^9^, have shown increases in the G:L ratio following capture, which can persist for at least 72 h. It is unknown how the G:L ratio characterises or responds to stress in reproductively-active female chondrichthyans or their offspring.

We currently have very limited knowledge on how capture stress may influence reproduction in chondrichthyans. Viviparous chondrichthyans are probably more vulnerable to reproductive disturbance than oviparous species because of their greater energetic requirements needed to maintain the embryonic developmental environment. Further, their relatively long gestation periods increase the probability that they will be pregnant when captured. In this context, the aim of our study was to investigate the reproductive consequences of fisheries capture during pregnancy in a viviparous chondrichthyan species. To achieve this, we trawled pregnant female southern fiddler rays, *Trygonorrhina dumerilii*, and measured changes to maternal body mass (BM; inclusive of embryonic contributions during pregnancy) and both G:L ratio and sex steroid responses for up to 28 days following capture. Neonates were measured for size (total length (TL) and BM) and G:L ratio at birth. By determining sex steroid and immune responses to capture stress in pregnant females, we aim to understand how stress may affect both maternal and offspring fitness, which in turn can influence population structure, population dynamics and the ecological consequences of fisheries capture.

## Results

### Maternal TL and BM

Females gave birth within a similar time frame to that reported by Marshall *et al*.^36^; with the first observed birth occurring on April 8 (12 days after day 28) and the last on May 24, 2013. Although 10 dead embryos were extracted from four females that had died on May 25 (as noted in *Methods* section below), parturition was assumed to be imminent as indicated by the females’ distended cloaca. With a total of 41 neonates (including two under-developed still-born), females from the control and trawled groups carried a mean of 2.3 and 2.0 embryos, respectively.

Maternal TL and BM at collection from the wild ranged 880–1030 mm and 4.12–6.2 kg for females allocated to the control group, and 900–988 mm and 4.02–5.9 kg for females allocated to the trawl group. There was no significant difference in either TL (ANOVA: F_1,17_ = 0.833, p = 0.374; Table 1) or BM (ANOVA: F_1,17_ = 2.14, p = 0.162; Table 1) at time of collection between trawled and control groups. Trawling significantly reduced post-partum BM relative to control animals (ANOVA: F_1,16_ = 6.411, p = 0.022; Table 1) and all females giving birth in May were significantly heavier at the time of parturition than those giving birth in April (ANOVA: F_1,16_ = 4.921, p = 0.041; Table 1).

### Maternal immune and sex steroid responses

**Figure 1:**
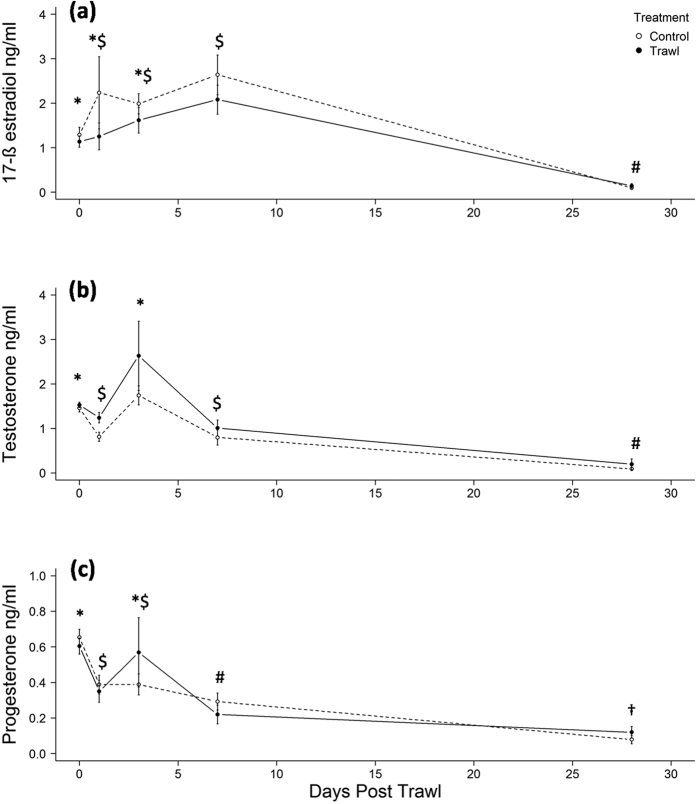
(**a**–**c**) Maternal sex steroid concentrations between trawled (n = 9) and control (n = 10) females spanning up to 28 days post trawling. *^$#†^Significant (p = <0.05) differences exist across time periods only.

Trawling did not significantly affect any sex steroid concentrations during pregnancy (LME; E_2_: F_1,17_ = 1.015, p = 0.328; T: F_4,72_ = 4.214, p = 0.056; P_4_: F_1,17_ = 0.068, p = 0.797; Fig. 1a–c) and in all females there were significant changes in all sex steroid concentrations over time between day 0–day 28 (LME; E_2_: F_4,72_ = 65.692, p = <0.001; T: F_4,72_ = 16.167, p = <0.001; P_4_: F_4,72_ = 14.960, p = <0.001; Fig. 1a–c). Post hoc paired t-test comparisons revealed that E_2_ continued to rise after capture, peaking significantly higher on day 7 than day 0, but then declined significantly to a level below that on day 0 by day 28 (Fig. 1a). Post hoc paired t-test comparisons revealed that T and P_4_ initially declined by day 1 before increasing to their respective peaks on day 3 (Fig. 1b,c) and then significantly declined again to a level below that of day 0 by day 28 (Fig. 1b,c).

**Figure 2:**
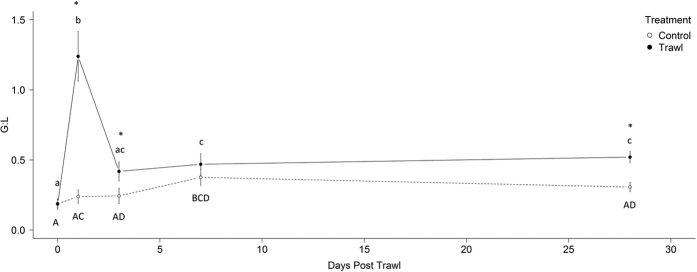
Maternal granulocyte to lymphocyte ratio (G:L) response to trawling (n = 9) and control (n = 10) treatments spanning up to 28 days post trawling. *Significantly different (p = <0.05) compared to respective control. ^abc^Significant differences across time *within* trawled females (p = <0.05). ^ABC^Significant differences across time *within* control females (p = <0.05).

The G:L ratio was significantly affected by an interaction between treatment and time (LME: F_4,68_ = 13.861, p = <0.001; Fig. 2). Post hoc analyses of treatment contrasts revealed that trawled females had a significantly elevated G:L ratio compared to control females at day 1, day 3 and day 28 but were not significantly different at day 7 (Fig. 2). Within group differences determined by post hoc paired t-test comparisons revealed that the G:L ratio of trawled females reached its peak by day 1 (Fig. 2), after which it declined by day 3 but remained above the initial value until day 28 (Fig. 2). In contrast, the G:L ratio of control females was not significantly elevated until day 7, at which point it was similar to trawled females, and began to return day 0 levels by day 28 (Fig. 2).

### Neonatal TL, BM, and G:L ratio

Neonatal TL was significantly affected by an interaction between month of parturition and maternal treatment group (ANOVA: F_1,34_ = 18.190, p = <0.001, Table 1). Post hoc comparisons showed that neonates from trawled mothers had significantly shorter TLs compared to control neonates in April (F_1,34_ = 47.290, p = <0.001, Table 1) but not in May (F_1,34_ = 1.549, p = 0.222, Table 1). Trawling significantly reduced neonate BM (ANOVA: F_1,35_ = 26.110, p = <0.001, Table 1) irrespective of month of birth (ANOVA: F_1,35_ = 2.368, p = 0.133, Table 1). Neonate sex did not affect either TL (ANOVA: F_1,34_ = 2.949, 0.095) or BM (ANOVA: F_1,35_ = 3.631, 0.065). Only 14 neonates could be sampled for their G:L ratio response due to the difficulty in extracting blood; nine neonates from trawled mothers exhibited a mean G:L ratio of 2.95 (±0.82) which was significantly higher than the mean G:L ratio of 1.12 (±0.14) exhibited by five neonates from control mothers (Mann-Whitney-Wilcoxon: W = 6, p = 0.029).

### Observations

From our visual observations, trawled females frequently exhibited a much less vigorous response to the presence of food than control females. At the end of any given feeding session, trawled females had more food remaining in comparison to control females. Control females also fed more quickly and there was frequently very little or no food remaining. This behaviour continued for eight weeks post-trawl, although the effect seemed to weaken gradually.

## Discussion

Our results indicate that capture during pregnancy in *T. dumerilii* caused stress that resulted in significantly reduced maternal post-partum BM and neonatal size (TL and BM) relative to untrawled control animals. A persistent elevation in the G:L ratio of trawled females suggests further energy constraints on immune function, increasing the energetic cost of self-maintenance (as shown by reduced post-partum BM), reducing reproductive investment^12^,^31^ and resulting in neonates of smaller size. It remains unclear how circulating sex steroids affected reproductive investment in trawled females since there were no significant differences in E_2_, T or P_4_ when compared with control females. Neonates from trawled mothers also exhibited a higher G:L ratio, which may reflect not only alterations to immunological development *in utero* as a result of prenatal stress^37^, but also increased energetic costs of maintaining immune function and thus limiting their growth when exposed to pathogens *in utero*^38^,^39^. Therefore, our results show that the acute stress of a single capture during pregnancy can induce longer-term stress responses and influence the reproductive outcome for the animal captured.

Trawled *T. dumerilii* showed a sharp rise in G:L ratio that remained elevated for the duration of the experiment and may have further strained energy allocated to reproduction and self-maintenance. The increase in G:L ratio was probably in response to the air exposure component of trawling^9^ and may also be related to the increased cost to mount an immune response during pregnancy, because energy allocated to immune function is often diverted towards the greater energetic demands of reproduction^29^,^32^,^40^,^41^. Elevations of sex-steroids associated with reproduction were also found to be immunosuppressive and affected leukocyte proliferation in female little skates, *Leucoraja erinacea*^42^. We observed apparent reductions in food uptake following capture in trawled females for up to three weeks, which may have further limited energy available and contributed to a persistently elevated G:L ratio. We think that a connection between feeding and G:L ratio is likely because of the more general effects of nutritional state on immunocompetence^43^,^44^, and under nutrient-limited conditions during reproduction immune function can be impaired^31^. Although we did not quantify food intake, the mass change in trawled females was consistent with the reduced appetite and food intake seen in teleost species subjected to acute stress^45^,^46^,^47^,^48^. Therefore, an increased strain on energetic resources allocated to both immune and reproductive functions may have also contributed to the reduction in maternal post-partum BM that we measured. Reduction in body mass is considered a reliable biomarker of chronic stress in animals^33^.

Determining the effect of trawling on sex steroid concentrations is difficult because sex steroid concentrations in wild *T. dumerilii* are unknown. However, when comparing our results with those of the Australian sharpnose shark, *Rhizoprionodon taylori*, a species sharing a similar reproductive strategy of embryonic diapause^23^, sex steroid concentrations in *T. dumerilii* were markedly lower. By day 28, concentrations of E_2_, T and P_4_ were respectively lower compared to *R. taylori* by approximate magnitudes of 16, 28 and 7 times, potentially indicating the suppression of sex steroid synthesis due to chronic stress^30^. However, the effects of chronic stress are likely a result of captivity given the same response was evident in the control group. Interestingly, transient increases in sex-steroids resulting from the relatively mild but repeated stress of handling (day 0–day 7) may have caused premature ovulation in pregnant *T. dumerilii*^49^,^50^. However, this could be coincidental because increases in sex steroids associated with ovulation during late-gestation also occur in *R. taylori*^23^. Because sex steroid concentrations can vary depending on reproductive modes and species^20^, documenting species-specific cycles in sex steroid concentrations of wild-caught females are necessary in future studies to better understand the effects of captivity and experimental conditions when assessing the potential implications of fisheries capture.

Prenatal stress of our trawled animals reduced neonate size, which may be related to increased embryonic exposure to maternal glucocorticoids^51^,^52^,^53^,^54^. Glucocorticoids released in response to stress are initially adaptive, by promoting rapid mobilisation and utilisation of energy favouring immediate survival, but chronic elevations can incur energetic costs and potentially limit growth^55^. In our study animals, increased circulating glucocorticoid concentrations associated with trawling may have permeated the egg capsule, within which the embryo develops until shortly before parturition, and influenced the neonate’s ability to convert nutrients to growth. Although the presence of glucocorticoids in the intrauterine environment of *T. dumerilii* were not determined, increased vascularisation of the uteri may have promoted the diffusion of glucocorticoids from the blood stream^56^,^57^. Egg capsules in smooth dogfish, *Mustelus canis*^58^, and bonnethead shark, *Sphyrna tiburo*^59^, are permeable to molecules up to 1355 and 6000 Da respectively, which could permit entry of a glucocorticoid such as corticosterone measuring 346 Da. Egg capsule thickness varies with both the species and the time of gestation^59^ and we did not measure capsule thickness in *T. dumerilii*, but the capsules of viviparous species are thin compared to oviparous species and are likely to be sufficiently permeable to enable entry of steroid molecules^59^.

Reductions in neonate size we measured may also be attributed to trawled mothers being restricted in their capacity to supplement embryonic nutrition. Although not yet confirmed in *T. dumerilii*, most viviparous species, particularly batoids, are thought to provide additional maternal nutrients in the form of histotroph (a uterine secretion)^60^, the degree of which affects the embryos’ reliance on yolk^61^. Increased constraints on the maternal energy budget as a result of trawling, as implied by reduced post-partum BM and elevated immune responses, may have restricted the production of histotroph. Given that yolk quantity is finite and all neonates exhibited a macroscopically visible internal yolk sac, lower BM in neonates from trawled mothers may therefore indicate an increased reliance on yolk *in utero*. Like the internal yolk sac, energy reserves in dusky shark, *Carcharhinus obscurus*, neonates take the form of an enlarged liver (contributing up to ~20% of neonate BM) that can vary in mass depending on maternal provisioning of additional resources as dictated by maternal body condition^62^.

Prenatal stress increased the G:L ratio response in neonates, which may also be related to increased exposure to maternal glucocorticoids. Acute elevations of glucocorticoids, particularly corticosterone, can stimulate immune responses, but sufficiently elevated glucocorticoid levels can be immunosuppressive^19^,^63^. Studies investigating embryonic and neonatal immune responses during pregnancy are limited to mammalian species, in which stress during the later stages of pregnancy has a profound and long-lasting impact on the offspring’s immunological development and function^37^,^64^. In two viviparous elasmobranch species, the nurse shark, *Ginglymostoma cirratum*, and dwarf ornate wobbegong, *Orectolobus ornatus*, the embryonic immune system is also thought to undergo extensive development during late gestation/pre-partum stages because of the passive transfer of maternal immunity and exposure to seawater during flushing of the uterus^38^,^65^. Thus, *T. dumerilii* embryos exposed to prenatal stress may have been immunologically challenged when both the egg capsule was broken and the uteri were flushed, exposing them to water-borne pathogens. Immune challenges associated with water-borne pathogens *in utero* may have also influenced energy allocation to growth and thus influenced neonate size^39^. We suggest our results regarding neonatal immune responses be interpreted with some caution due to the small sample size but nonetheless there is evidence for a link between the maternal immune status and regulation of the developmental environment *in utero*, thereby affecting embryonic and neonatal immune responses to prenatal capture stress.

Prenatal stress can potentially affect population recruitment by influencing neonatal survival, development and fitness. Larger body sizes at birth are generally considered advantageous^66^ as size-selective predation may be more intense for smaller neonates^67^ and lower energy reserves (internal yolk sacs or liver) may increase the risk of starvation resulting from poor foraging skills or low prey abundance early in life^68^. The immune response of neonates is difficult to interpret as it can be unclear whether an elevated response is indicative of an active or challenged system^35^. Nonetheless, maladaptive immune responses may include increased vulnerability to pathogens and reduced growth rate, the latter potentially affecting size-related fecundity^62^,^69^.

The maternal response to capture stress during pregnancy may influence several aspects relating to future reproductive success. Decreases in overall body condition may be reflective of lower lipid stores limiting maternal energy reserves and reductions in egg size or number^13^,^26^,^31^, which ultimately determine resources available for embryonic growth. Females with lower body condition may miss mating opportunities if they require longer than normal periods to regain energy stores before the next reproductive bout. This may be particularly important for species such as the scalloped hammerhead, *Sphyrna lewini*^70^, sharpnose shark, *R. taylori*^23^ and smooth dogfish, *M. canis*^71^, which breed annually and mate shortly after parturition.

The influence that capture stress has on sex steroid responses in pregnant females is not clear, but their potential suppression could influence hormonal composition and overall quality of follicles. In *S. tiburo* the maternal deposition of hormones in yolk is correlated with circulating concentrations during vitellogenesis and may influence aspects such as embryonic growth and sex differentiation^72^. Estradiol regulation of yolk synthesis by the liver may also be affected, influencing the quality of yolk available during early embryonic development^20^. Furthermore, declines in E_2_ have been suggested as possible causes of lower fertility rates in chondrichthyans by impairing sperm storage^73^.

Immune challenges during pregnancy may also affect yolk composition and embryonic development. Maternal immune factors have recently been shown to be passively deposited in follicles of *G. cirratum*^65^, which are likely to affect the immunological development of offspring^44^. Little is known about how yolk composition affects embryonic development in chondrichthyans^65^,^72^ and maternal effects that determine yolk composition should be examined further, given the potential for capture stress to have long-lasting intergenerational effects.

Predicting population-level responses to prenatal stress is difficult because the reproductive outcomes are likely to vary both across reproductive strategies and also between species within a given reproductive strategy. Compared to oviparous species, viviparous species are likely to be most vulnerable to reproductive disturbance, particularly those whose breeding habitats overlap with both commercial and recreational fisheries. Symptoms of stress observed in our study could therefore manifest in fisheries where frequent recaptures are possible, particularly in species that can exhibit high site-fidelity^74^,^75^. For example, *T. dumerilii* frequently occupy areas in Port Phillip Bay that are extensively fished by both commercial and recreational hook-and-line fishers and it is not uncommon for repeat captures of an individual animal to occur within hours or weeks (L. Guida *pers. obs*.).

The sub-lethal effects of fisheries capture on reproductive effort and success requires significantly more attention, given the potential for intergenerational effects. Neonatal survival and growth may be compromised by reduced size at birth and potentially altered immune function. By identifying the vulnerability of species and populations to reproductive disturbance, we can improve our understanding of population structure and dynamics in response to fishing pressures.

## Methods

### Ethics

This study was conducted in accordance with relevant animal use legislation. Experimental protocols were approved by a Monash University Animal Ethics Committee (approval number BSCI/2012/16), Fisheries Victoria (permit number RP1115) and Parks Victoria (permit number 10006544).

### Study species

*Trygonorrhina dumerilii* is an abundant species endemic to southern waters of Australia and is frequently caught as bycatch in trawl fisheries^76^. Ovulation is thought to occur in April–May, after which diapause commences and embryonic development is delayed for a period of 7‒8 months^36^. In December diapause ends and embryos recommence development with rapid growth until parturition during April‒May. Diapause delays gestation until favourable environmental conditions are present and is also thought to assist with maternal energy requirements throughout reproduction^23^,^77^.

### Animal collection and husbandry

Nineteen pregnant *T. dumerilli* were collected by hand using SCUBA from Swan Bay, Victoria, Australia (38°25’S 144°67) from 15‒20^th^ February, 2013. Pregnancy was determined by presence of embryos detected by ultrasound (L6.2 linear transducer probe at 8–5 MHz, Ibex Pro Portable Ultrasound, *E. I. Medical Imaging*, USA). All females were transported by a boat, which contained a tank with continually-flowing ambient seawater, to tanks at a research facility situated adjacent to Swan Bay. Collection and transportation of females to tanks was completed within 2 h on the day of collection. All females were randomly assigned to either a trawl (n = 9 animals) or control (n = 10 animals) treatment group, with each group housed in a separate 19,000 L circular tank supplied with constantly flowing seawater at ambient temperatures ranging from 24 °C at the time of the animals’ initial collection from the wild in February to 17 °C at the study’s conclusion in May.

Animals were left to acclimatise to captivity for 7‒12 days prior to experimentation. Once placed in tanks, all females were treated identically and were first fed between 24 and 48 h after capture. They were fed 5% of BM twice per week with a diet of two-thirds commercially available Australian sardines (*Sardinops neopilchardus*) and one-third prawns (species unknown). All females were fed at the same time and uneaten food was removed from the tank after 3 h.

### General experimental design

To describe maternal and neonatal responses to trawling and subsequent air exposure (henceforth, ‘air exposure’ is implicit when referring to trawling) during pregnancy, all pregnant females were regularly blood-sampled to monitor changes in BM, sex steroid concentrations (E_2_, P_4_, T) and G:L ratio immediately before trawling (day 0) and at 1, 3, 7 and 28 days post trawling. At parturition, neonates were measured for TL, BM and G:L ratio. In the context of this study, ‘at parturition’ refers to the moment in time at which the neonate was first observed to be present in the tank. Although it was not possible to estimate exactly how long after parturition the neonates were measured, it was within a maximum of 16 hours. Nine pregnant females were placed together in a single, stationary cod-end (monofilament net bag with stretched mesh diameter of 10.2 cm and bag length 110 cm) and trawled for 8 h. Although 4‒6 h is more common in trawl fisheries^78^, our trawl duration is within the range of commercial practice and was chosen to compensate for the absence of stressors such as packing/crowding, which occur in commercial capture. The cod-end was placed in a 19,000 L tank that had a large rotating paddle measuring 2.0 × 1.0 m (W × H) in the centre driving a water current of ~0.6 m/s in front of the cod-end (see Frick *et al*.^78^ for detailed description of trawl design and function). Once trawling had ceased, females were immediately placed together in an empty tub measuring 2.0 × 1.0 × 0.7 m (L × W × H) to undergo air exposure for 30 min in order to simulate the commercial process of sorting the catch once landed. The pregnant females were returned to their housing tank after air exposure. The control group did not undergo trawling and air exposure but experienced identical handling, external measurement and blood sampling protocols throughout the experiment.

To quantify maternal sex steroid concentrations and G:L ratios, all pregnant females had 2 ml of blood sampled by caudal venipuncture using heparinised syringes fitted with 18-gauge needles. Blood sampling occurred prior to any ultrasounds and external measurements. On each occasion, sampling took less than 30 s at capture. After extraction, blood smears were taken to measure G:L ratio and the remaining blood was centrifuged (HaematoSpin 1400, *Hawksley*, Sussex, UK) for 5 min at 10,000 rpm. The plasma was collected and stored at −20 °C until it was thawed for the analysis of sex steroids.

Maternal TL (mm) and BM (kg) were measured on initial capture in the wild and BM was again measured once the animal was identified as being post-partum, to account for embryonic contributions to maternal BM. All individuals were monitored in tanks for signs of parturition and confirmation of post-partum status was determined by ultrasound when neonates were first observed in tanks. Unfortunately, the day before routine tank inspection for neonates, eight females from the control group died due to a water supply malfunction. Four dead females were still pregnant and their post-partum BM was estimated by weighing their carcass before and after the removal of neonates, which were also measured for TL (mm) and BM (g).

Live neonates had blood samples (up to 200 μl) taken once at birth by caudal venipuncture with heparinised syringes fitted with 21-gauge needles. To minimise stress, blood was only drawn from neonates if it could be completed within 30 s. Blood could only be obtained from 14 of 41 neonates (trawled = 9, control = 5) and their respective blood-smears were prepared to calculate the G:L ratio at a later date. Following blood extraction, TL and BM were measured. We also noted the presence of internal yolk sacs visible though the body wall of neonates. Because of the death of some pregnant females as noted above, it was not possible to determine how trawling affected the timing of birth in those females. Due to permit and ethics requirements, neonates were released immediately into the wild following initial sampling.

### G:L ratio and sex steroid analysis

**Figure 3:**
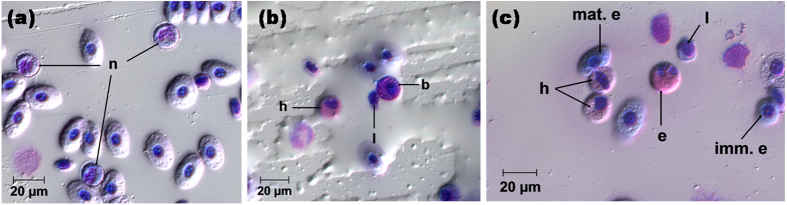
(**a**–**c**) Blood cell types seen at x400 magnification using blood smears stained with May-Grünwald and Giemsa solutions. *Mature erythrocyte (mat. e*): elliptical in shape, with a central nucleus and a low nucleus to cytoplasm ratio. *Immature erythrocyte (imm. e*): Rounder in shape than mature counterparts with a high nucleus to cytoplasm ratio. *Lymphocyte (l*): Dark blue/purple, often indented nucleus with a high nucleus to cytoplasm (stains light blue) ratio. *Neutrophil (n*): Segmented and/or multi-lobed nucleus usually centrically located with light blue-grey granules in a light purple cytoplasm. *Heterophil (h*): Eccentric, lobed or banded nucleus with a hazy, pink-red granulated cytoplasm. *Eosinophil (e*): Generally larger than heterophils with an eccentric, banded nucleus and intense/bright red staining of distinct ‘rod’ shaped granules. *Basophil (b*): Round cells with a round, eccentric, dark blue/purple nucleus. Often obscured by dense, black and purple granulation in light blue-grey cytoplasm. Degree of granulation can vary due to stain-induced degranulation. In some cases cytoplasm appears sparsely granulated.

To quantify the G:L ratio, blood-smear slides were prepared from each sample obtained from females and from each neonate. Blood-smears were first air-dried in sealed containers for 2–8 h before fixing in methanol for 10 min. Fixed slides were stained with May-Grünwald (solution diluted 1:1 with water; *Australian Biostain*, Traralgon, Victoria, Australia) and Giemsa (solution diluted 1:9 with water; *Australian Biostain*) for 15 min each before rinsing three times in distilled water, followed by standing in fresh distilled water for 5 min. Slides were examined using compound microscopes at ×400 magnification and leukocyte counts were made using the technique described by Van Rijn and Reina^8^. A minimum of 250 granulocytes (neutrophils, heterophils, eosinophils and basophils) and lymphocytes were identified per slide. Counting ceased once all cells had been identified in the final field of view. The identification of leukocytes (Fig. 3) was based on the descriptions found in Van Rijn and Reina^8^, Claver and Quaglia^79^, Clauss^80^ and Tripathi *et al*.^81^. The accuracy of leukocyte profiling and the G:L ratio was tested by randomly resampling a subset of eight slides (with their identity concealed) and comparing their individual G:L ratio recount to their original counts. To test for any learning effect when identifying cells, the counts from the first seven slides counted on day 0 were compared against the last seven slides counted on day 0. A potential learning effect and the accuracy of leukocyte counts were both determined using a log-transformed paired t-test. Results indicate that there was no significant learning effect (t_10.835_ = 0.233, p = 0.820) and that differential counts were consistently accurate (t_7_ = −0.8008, p = 0.450).

Plasma concentrations of T, E_2_ and P_4_ were measured by radioimmunoassay (RIA). Testosterone antiserum was purchased from Novus Biologicals © (USA), and reconstituted by diluting 1:10 in Phosphate Buffered Saline (PBS) assay buffer containing 0.1% of gelatin and 0.01% of thiomersal. Progesterone and E_2_ antisera were purchased from Sigma-Aldrich (Australia) and were reconstituted by adding 5 ml of Tris buffer (pH 8, 0.1 m HCl). Tritiated T [1,2,6,7-^3^H], E_2_ [1,2,6,7-^3^H] and P_4_ [1,2,6,7-^3^H] were purchased from Perkin-Elmer (Australia) and 50 μl of each was diluted in 5 ml of 100% ethanol and kept as separate stocks for the assay. For each respective hormone assay, plasma samples (200 μl) were extracted twice with ethyl acetate (1 ml), and two 100 μl aliquots were transferred to assay tubes. Samples were then evaporated by air and 100 μl of PBS assay buffer was added to reconstitute each extract. Duplicate standards (of T, E2 and P4 in ethanol) ranging from 0–800 pg per tube were then evaporated under air and reconstituted in assay buffer. Reconstituted antiserum (100 μl) and 50 μl of tritiated stock were then added to each assay tube and incubated overnight at 4 °C. Bound and free fractions were then separated using dextran-coated charcoal and aliquots of the supernatants counted in a Beckman LS 5801 liquid scintillation counter. All assays were validated by the evaluation of the slope of serially diluted extractions of plasma against the assay standards. Overall extraction efficiency was determined for each hormone by adding 20 μl of the appropriate ^3^H-labelled steroid to 200 μl pooled aliquots of plasma and measuring the radioactivity that remained following extraction and reconstitution. Extraction efficiencies were 93% (T), 87% (P_4_) and 82% (E_2_), and final assay values were corrected accordingly. The detection limit for all assays was 0.05 ng (ml plasma)^−1^. Intra- and inter-assay variability was determined by including in each assay replicates of three levels of commercially available human control serum (CON4, CON5 and CON6 DPC). Inter-assay variability was 8% (T), 9% (E_2_) and 11% (P_4_) and intra-assay variability was <5% for all hormones.

### Statistical analyses

One-way ANOVAs were used to test for significant differences between groups in the response variables of TL and BM at initial capture from the wild. A factorial ANOVA was used to determine the effect of treatment on post-partum BM with respect to the month of parturition.

Change in concentration of each sex steroid and in G:L ratio were tested using a linear mixed effect models (LME) whereby treatment was the main factor, individual females were the blocking factor and time was the sub-factor. For these analyses, values of sex steroid concentrations and G:L ratio were log-transformed to ensure residuals were normally distributed. Paired t-tests with Holm-Sidak corrected p-values were used to compare differences in time within treatment groups for all sex steroids and G:L ratio. Neonatal G:L ratio from trawled and control mothers were compared using a Mann-Whitney-Wilcoxon t-test due to violations of normality. Sample numbers of neonatal G:L ratio were limited due to the difficulty extracting blood from neonates. Neonates were selected from differing birth dates to avoid possible sibling-confounding effects on the G:L ratio.

Because different females could give birth simultaneously during unobserved periods, it was not always possible to identify the mother of each neonate. Thus, neonates were pooled by their respective maternal treatment group in all analyses. The effects of treatment, month of birth, and sex of neonate on each of BM and TL were tested by factorial ANOVAs.

The most parsimonious models for each test by LME and ANOVA are presented in our study and were determined by step-wise backward elimination of non-significant interaction terms from the full model. Any significant interactions were investigated by analysis of main effects. All analyses were performed using R 2.15.3 software package^82^ using a significance value of p < 0.05.

## Additional Information

**How to cite this article:** Guida, L. *et al*. Prenatal stress from trawl capture affects mothers and neonates: a case study using the southern fiddler ray (*Trygonorrhina dumerilii*). *Sci. Rep.* **7**, 46300; doi: 10.1038/srep46300 (2017).

**Publisher's note:** Springer Nature remains neutral with regard to jurisdictional claims in published maps and institutional affiliations.

## Figures and Tables

**Table 1:** Reported means ± standard error of both maternal and neonatal body mass (BM) and total length (TL) with respect to treatment.

Treatment group	Time			
	Initial capture (wild)	April (postpartum)	May (postpartum)	
Maternal				
*Control (n* = *10*)				
BM (kg)	5.24 ± 0.21	5.05 ± 0.16	5.89 ± 0.14^#^	
TL (mm)	955 ± 13	—	—	
*Trawled (n* = *9*)				
BM (kg)	4.81 ± 0.20	4.62 ± 0.09*	5.03 ± 0.07*^#^	
TL (mm)	940 ± 9	—	—	
Neonatal				
*Control mothers (n* = *22*)				
BM (g)	—	118 ± 4	120 ± 4	
TL (mm)	—	270 ± 3	263 ± 2	
*Trawled mothers (n* = *17*)				
BM (g)	—	86 ± 7*	100 ± 7*	
TL (mm)	—	238 ± 3*	258 ± 7	

*Significantly different (p = <0.05) compared to respective control. ^#^Significant differences (p = <0.05) between postpartum months within treatments.
